# Supplementary material for: Extreme Weather Events Enhance DOC Consumption in a Subtropical Freshwater Ecosystem: A Multiple-Typhoon Analysis
Source: Microorganisms. 2021 Jun 1;9(6):1199. doi: 10.3390/microorganisms9061199 (PMC8230144; doi:10.3390/microorganisms9061199)
Supplement: Supplementary file 1 [file microorganisms-09-01199-s001.zip › microorganisms-1218814-SI.pdf]

microorganisms

Table S1. The name, visiting period, total rainfall, and maximal wind speed of the typhoons that had swept through Taiwan during the period of 2004~2009. The two typhoons (2004 KOMPASU and 2008 NURI) that had almost no precipitation were not included in our analysis.

Year	Name	Period	Total Rainfall[@] mm	Max wind speed M s^{-1}
2004	CONSON	06/07～06/09	40	33
	MINDULLE	06/28～07/03	291	45
	KOMPASU	07/14～07/15	2	20
	RANANIM	08/10～08/13	205	40
	AERE	08/23～08/26	541	38
	HAIMA	09/11～09/13	465	18
	MEARI	09/26～09/27	213	40
2005	HAITANG	07/16～07/20	488	55
	MATSA	08/03～08/06	410	40
	SANVU	08/11～08/13	71	25
	TALIM	08/30～09/01	377	53
	KHANUN	09/09～09/11	129	43
	DAMREY	09/21～09/23	394	25
2006	EWINIAR	07/07～07/09	28	51
	BILIS	07/12～07/15	188	25
	KAEMI	07/23～07/26	57	38
	SAOMAI	08/09～08/10	146	48
	BOPHA	08/07～08/09	152	25
	SHANSHAN	09/14～09/16	161	48
2007	PABUK	08/06~08/08	182	28
	WUTIP	08/08~08/09	94	18
	SEPAT	08/16~08/19	347	53
	WIPHA	09/17~09/19	298.2	48
2008	KALMAEGI	07/16～07/18	75	33
	FUNG-WONG	07/26～07/29	263	43
	NURI	08/19～08/21	0	40
	SINLAKU	09/11～09/16	782	51
	HAGUPIT	09/21～09/23	19	45
	JANGMI	09/26～09/29	457.4	53
2009	LINFA	06/19～06/22	22	28
	MOLAVE	07/16～07/18	35	30
	MORAKOT	08/05～08/10	344	40

@, accumulated precipitation during the typhoon period.
